# Low Power Compact 3D-Constructed AlScN Piezoelectric MEMS Mirrors for Various Scanning Strategies

**DOI:** 10.3390/mi14091789

**Published:** 2023-09-19

**Authors:** Jeong-Yeon Hwang, Lena Wysocki, Erdem Yarar, Gunnar Wille, Fin Röhr, Jörg Albers, Shanshan Gu-Stoppel

**Affiliations:** Fraunhofer Institute for Silicon Technology (ISIT), Fraunhoferstraße 1, 25524 Itzehoe, Germany; jeong-yeon.hwang@isit.fraunhofer.de (J.-Y.H.);

**Keywords:** MEMS mirror, aluminum scandium nitride (AlScN), piezoelectric actuation, quasi-static scan, resonant scan

## Abstract

In this paper, the newly developed 3D-constructed AlScN piezoelectric MEMS mirror is presented. This paper describes the structure and driving mechanism of the proposed mirror device, covering its driving characteristics in both quasi-static and resonant scan modes. Particularly, this paper deals with various achievable scan patterns including 1D line scan and 2D area scan capabilities and driving methods to realize each scanning strategy. Bidirectional quasi-static actuation along horizontal, vertical, and diagonal scanning directions was experimentally characterized and even under a low voltage level of ±20 V, a total optical scan angle of 10.4° was achieved. In addition, 1D line scanning methods using both resonant and non-resonant frequencies were included and a total optical scan angle of 14° was obtained with 100 mV_pp_ under out-of-phase actuation condition. Furthermore, 2D scan patterns including Lissajous, circular and spiral, and raster scans were realized. Diverse scan patterns were realized with the presented AlScN-based MEMS mirror device even under a low level of applied voltage. Further experiments using high voltage up to ±120 V to achieve an enhanced quasi-static scan angle of more than 20° are ongoing to ensure repeatability. This multi-functional MEMS mirror possesses the potential to implement multiple scanning strategies suitable for various application purposes.

## 1. Introduction

Microelectromechanical systems (MEMS) mirrors, also referred to as MEMS scanners and MEMS microscanners, feature a reflective surface with diameters ranging from a few hundred micrometers to a few millimeters [1]. These reflective micromirrors can have configurations such as circular [2,3,4,5], rectangular [6,7,8], or elliptical shape [9,10]. MEMS mirrors, with their reflective surfaces, rotate along one or two scanning axes, steering the incident laser beams to the desired and designated positions. In other words, MEMS mirrors can be utilized for not only providing but also acquiring information by illuminating the one-dimensional (1D) or two-dimensional (2D) area. Thanks to their scanning capabilities, MEMS mirrors have been utilized in diverse application fields, including projection display systems for augmented reality (AR) and virtual reality (VR) smart glasses [11,12,13], light detection and ranging (LiDAR) systems [3,4,7,10,14,15,16,17], and even bio-imaging applications [8,18].

The required specifications for MEMS mirrors have varied in accordance with the prevailing market demand of that era. MEMS mirrors were primarily used for the target application of projection display systems. Small apertures for MEMS mirrors were pursued, with a diameter ranging from 1 mm to 1.5 mm. The driving frequency typically ranged in the tens of kilohertz and it varied depending on the resolution of the desired display system [1,19,20,21]. Among various actuation principles, the relatively easy-to-fabricate electrostatic actuation method was predominantly utilized to realize the MEMS mirrors [22,23,24]. However, electrostatic actuation had its limitations in implementing high resolution display systems. Research initiatives were also conducted to employ piezoelectric actuation, which has the advantages of generating significant force and having a fast response time [9,25,26,27]. However, due to limitations in the fabrication technology, there were constraints on further development. Alternative research efforts were also tried to realize the MEMS mirrors for high resolution display systems by utilizing an electromagnetic actuation method [28,29,30,31]. However, remarkable achievements in the market were not apparent. Afterwards, as interest in autonomous driving increased, MEMS mirrors began to regain attention as an alternative technology to overcome the limitations of conventional mechanical LiDAR, such as bulkiness, high cost, low performance, and challenges in mass production [17]. With the shift in the target application to LiDAR, MEMS mirrors have been required to reflect relatively high-power lasers and transmit the reflected light or photons to detectors, necessitating mirror sizes of over 2 mm in diameter [4,32,33,34,35]. Additionally, the driving frequencies have decreased to the kilohertz range with a cost of large aperture. Furthermore, recently, there has been growing interest in utilizing MEMS mirrors for developing smart glasses, which were actively researched as projection display systems [11,12,13]. Especially, the advancement in piezoelectric thin-film fabrication processes has helped the entry of MEMS mirrors into the market.

In the context of trends in MEMS mirror research, another notable shift is related to the scan mode. While in the past, the focus was mainly on utilizing the resonant scan mode of MEMS mirrors, recently, the development of quasi-static MEMS mirrors has been increased for application fields requiring to hold the mirror’s tilting position [36,37,38]. That is, there is an increasing interest in the potential for new scanning modes in MEMS mirrors, beyond their typical scan modes. This interest encourages promising chances for applications in various fields related to beam steering and other previously unexplored areas, such as 3D printing technology and invasive measurement tools. In accordance with the recent research trends and market demands, this paper focuses on investigating the various scanning capabilities by utilizing our newly developed AlScN MEMS mirror devices.

**Figure 1:**
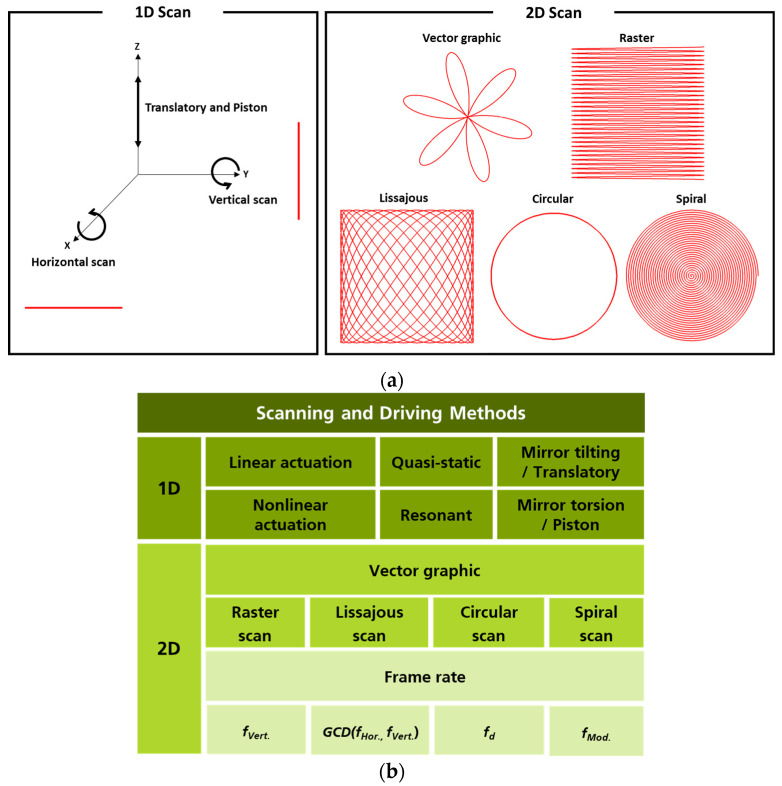
(**a**) Examples of capable 1D and 2D scan patterns realized by MEMS mirrors, (**b**) types and driving methods of various scan patterns.

Figure 1 shows a wide range of scan patterns that MEMS mirrors can achieve and how they enable the display or acquisition of 1D or 2D information. Each scan pattern has unique characteristics and advantages, leading to the selection of appropriate scan patterns depending on the specific application’s goals. As shown in Figure 1a, MEMS mirror scan patterns are mainly categorized into 1D and 2D scans, and it is typically related to the number of rotational axes that MEMS mirrors have. When the rotational axis is along the *x*-axis, the line scan mode is referred to as horizontal scan, and when it is along the *y*-axis, it is called vertical scan. The corresponding two scan modes of horizontal and vertical scan are named mirror tilting or torsion modes. There is a unique mode involving movement in the *z*-axis direction, known as translatory or piston mode. As shown in Figure 1b, 1D scan patterns vary depending on the driving methods, either quasi-static or resonant modes. The selection of driving method and scan pattern is closely related to a pursued application. For example, fiber-based frequency modulated continuous wave (FMCW) LiDAR has been developed using a point-to-point scanning method, employing a quasi-statically driven mirror tilting mode with linear actuation [39]. By utilizing the resonant driving method, named mirror torsion mode, the MEMS mirror’s performance can be enhanced by taking advantage of its high Q-factor mode. This mirror device can achieve high scanning speed and a wide scan angle even with minimal input, especially at low voltage or low power consumption. Using a 1D line scan generated by resonant driving, a 2D LiDAR system with a single photon avalanche diode (SPAD) is shown in [40]. A 1D line scan-capable MEMS mirror can also be combined with a motor system to realize a 3D LiDAR system [34]. Another example in the field of automotive LiDAR involves the development of a 1D resonant MEMS mirror with a total optical scan angle (TOSA) of 180° [10]. The piston mode is one of the resonant modes where the reflective mirror structure moves up and down along the *z*-axis at a specific frequency. This mode can be utilized to obtain optical path differences for Fourier transform infrared (FTIR) spectroscopy [41].

By utilizing a combination of 1D quasi-static or resonant driving, various 2D scanning patterns can be generated. This enables the creation of diverse scanning patterns with different characteristics and functionalities. A vector graphic is the combination of two quasi-static modes. It is a forced actuation where the amplitude of a DC signal, either voltage or current, is adjusted to tilt the mirror at a specific angle [42]. Raster and Lissajous scan patterns are currently the most widely used scanning patterns, particularly in two key applications: LiDAR and projection display systems. In the case of LiDAR, these patterns are dominantly employed to develop 3D LiDAR systems that acquire information from the surrounding environment. On the other hand, as projection display systems, raster and Lissajous scan patterns are utilized to provide information on AR/VR smart glasses, enabling interaction with the surrounding environment and offering additional data to the users. Compared to the previous raster and Lissajous scan patterns, circular and spiral scans are unique scan patterns and have special advantages. The circular pattern, when combined with special related optics, enables the implementation of a 360° rotation, making it possible to create an omnidirectional scanning LiDAR system [43,44,45]. Moreover, it can be utilized for laser material processing as shown in [46]. The spiral scan pattern, as an advanced pattern derived from the circular scan, can be utilized to implement a 3D LiDAR system capable of omnidirectional scanning [43,47]. Thanks to the capabilities of MEMS mirror, it has been applied in various fields in the past and continues to be utilized in diverse application fields today. In this paper, our newly developed and characterized 3D constructed piezoelectric quasi-static MEMS mirrors are presented which can be applied to various applications’ needs.

**Figure 2:**
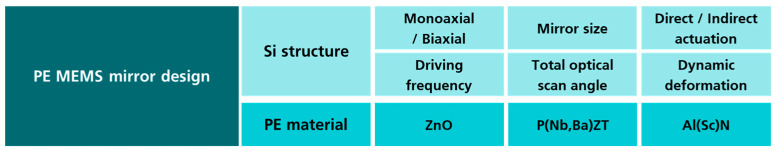
Key design considerations.

Figure 2 presents the key design factors needed to be considered when designing the piezoelectric MEMS mirror. The design parameters are mainly categorized into Si structure and piezoelectric (PE) material selection. The presented MEMS mirrors are biaxial scanners having two rotational axes and aluminum scandium nitride (AlScN) is utilized for piezoelectric actuation. Among various piezoelectric materials, AlN and AlScN are advantageous for piezoelectric actuation. In particular, AlScN thin film having wurtzite structure can have an enhanced piezoelectric coefficient by controlling the Sc concentration [48,49]. According to the previous research presented in [37], the piezoelectric constant (*e*_31_) of AlScN film can be improved twice compared to that of AlN. This result indicates that AlScN is a promising piezoelectric material for MEMS actuators and, thus, for MEMS mirror actuation. Moreover, the fabrication process is a complementary metal–oxide-semiconductor (CMOS) compatible with batch fabrication and enabling mass production. This piezoelectric material has linearity, enables bipolar actuation of contraction and expansion capable of generating strong actuation, and shows long-term stability [37]. In addition to its piezoelectric material advantages, the presented MEMS mirror device is compact as it is constructed in a 3D configuration compared to the conventional 2D planar structure. Based on our mature wafer stacking technology, the presented MEMS mirror is allowed to be fabricated with predetermined mirror plate sizes at the wafer level. Moreover, by fabricating separate components for the actuator part and mirror plate, it enables customized production with various mirror diameters to meet the demands of different application targets.

## 2. Proposed 3D-Constructed AlScN Piezoelectric MEMS Mirror

**Figure 3:**
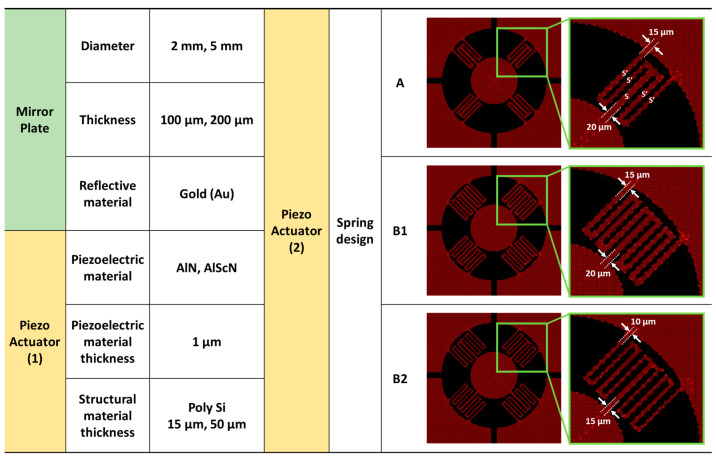
Detailed specifications and dimensions of 3D-constructed MEMS mirror devices. S and S’ represent the width of the serpentine spring.

**Figure 4:**
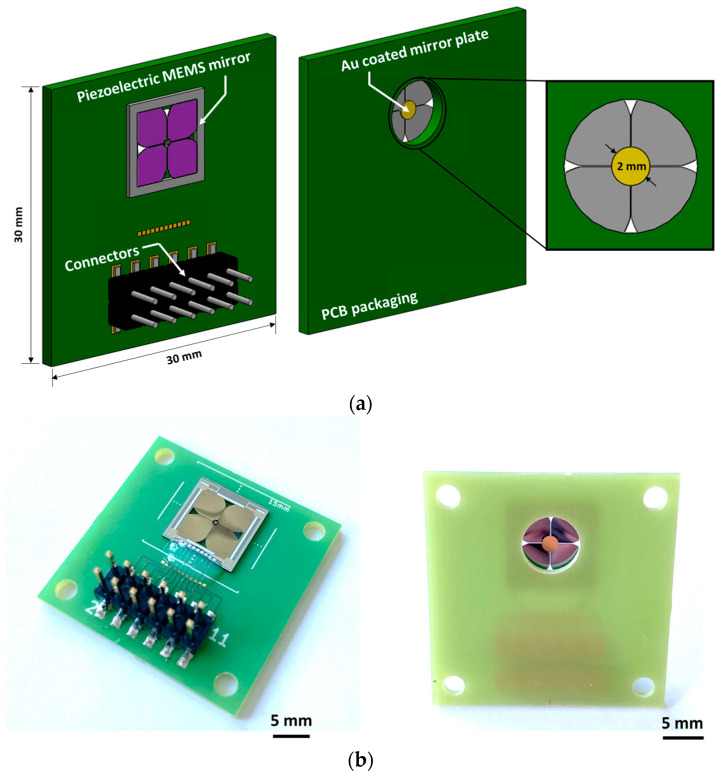
(**a**) Schematic of 3D-constructed piezoelectric MEMS mirror device and (**b**) photographs of fabricated and packaged devices.

**Figure 5:**
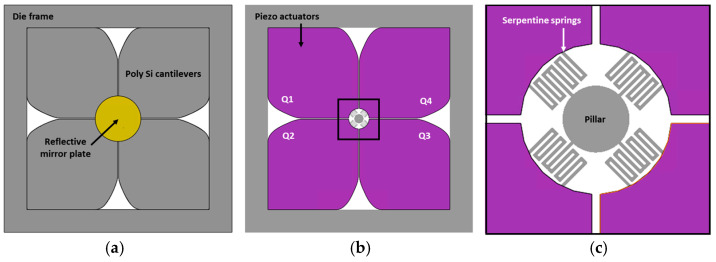
Planar views of designed piezoelectric MEMS mirror structure: (**a**) top side with mounted mirror plate, (**b**) back side of piezoelectric actuators, and (**c**) magnified view of serpentine spring structures.

Figure 3 shows the specification and dimensions of recently fabricated 3D-constructed MEMS mirror devices. The presented 3D-constructed piezoelectric MEMS mirrors are based on our previous design and the detailed fabrication process can be found in [37]. The utilized MEMS mirrors are shown in Figure 4. The size of the MEMS mirror die is 11 mm × 11 mm with a device footprint of 8 mm × 8 mm. The utilized diameter of the mirror plate is 2 mm and the reflective surface is coated with gold. The fabricated MEMS mirror die is mounted on the printed circuit board (PCB) with a dimension of 30 mm × 30 mm × 1 mm (length × width × thickness) and electrically connected by a wire bonding process. The schematic description of the developed biaxial MEMS mirror device is shown in Figure 5a,b. This MEMS mirror can be operated in both quasi-static and resonant driving. Four piezo actuators, shaped like rose leaves, are fabricated on a 15 μm-thick poly Si structural layer. These piezo actuators are coated with AlScN and are connected to a pillar structure via a serpentine spring structure. A gold-coated mirror plate is located on top of the pillar structure.

The utilized mirror plate thickness is 100 μm and it is sufficiently thick to guarantee the suppression of dynamic deformation that may occur during rapid rotation of resonant mode. Each piezo actuator is named from Q1 to Q4 in a counterclockwise direction as shown in Figure 5b. The serpentine spring structure connecting the piezo actuators and the pillar structure is shown in Figure 5c.

## 3. Driving Methods

**Figure 6:**
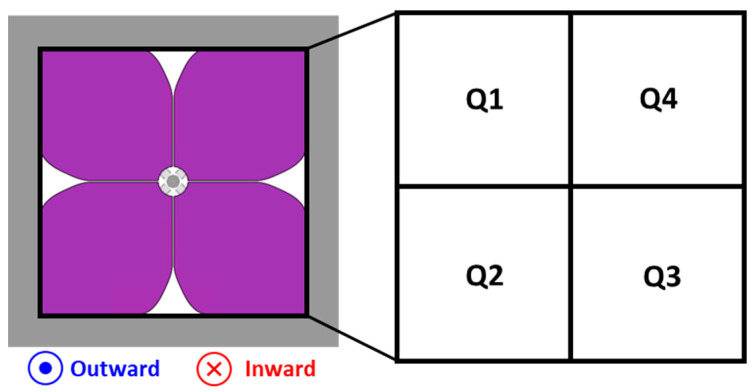
Configuration of four piezo actuators.

The developed 3D-constructed piezoelectric MEMS mirror can generate various scan patterns by utilizing both quasi-static and resonant scan mode. Thanks to the bipolar actuation capability of the utilized AlScN piezoelectric material, each piezo structure shown in Figure 6 can move either outward or inward depending on the polarity of the applied voltage signal. Moreover, various combinations of four actuators enable the realization of the multiple scan patterns previously shown in Figure 1a.

### 3.1. Driving Mechanism

**Figure 7:**
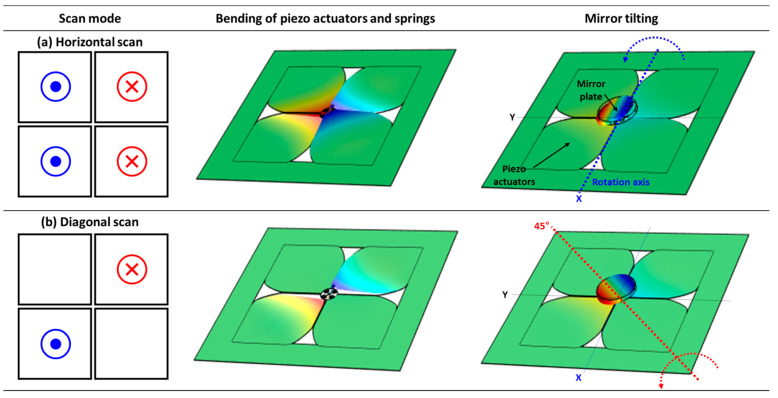
Finite element analysis results of (**a**) horizontal and (**b**) diagonal movement under ±20 V applied voltage on the piezo actuators.

Figure 7 presents the moving mechanism of the designed quasi-static MEMS mirror and COMSOL 6.1 finite element analysis (FEA) simulation tool was utilized to analyze the mirror device operations. Material properties were utilized from the material library in the simulation, while for piezoelectric materials, experimentally measured values were used. Figure 7 shows the static structural analysis with an applied voltage of ±20 V and the four outer surfaces of the die frame were fixed. A user-controlled mesh type was utilized to generate the fine mesh. Figure 7a shows the example of horizontal scan mode utilizing all four piezo actuators. When Q1 and Q2 are subjected to the same polarity of voltage, while Q3 and Q4 experience a voltage of the opposite polarity, the horizontal movement of the mirror can be maximized. According to Figure 7a, when the positive and negative voltages are applied to Q1, Q2 and Q3, Q4, respectively, Q1 and Q2 piezo actuators bend downward, whereas conversely, Q3 and Q4 bend upward. The serpentine spring structures connected to the piezo actuators also bend in the same direction, causing the mirror plate attached to the pillar to tilt horizontally. This enables the mirror plate to tilt along the horizontal direction, allowing for a precise tilting motion by adjusting and controlling the applied voltage level. Figure 7b describes the working mechanism of diagonal scan mode. For this case, positive voltage is applied to the Q2 actuator and negative voltage is applied to the Q4 actuator. As a result, Q2 bends downward while Q4 bends upward, and the mirror plate tilts along the rotational axis formed at the point where the horizontal and vertical axes intersect at a 45° angle. The two corresponding simulation results demonstrate that depending on the combination, the piezo actuators and springs bending vary based on which specific actuator is activated by the applied voltage and makes it possible to tilt the mirror plate in the desired direction.

### 3.2. Quasi-Static Driving

**Figure 8:**
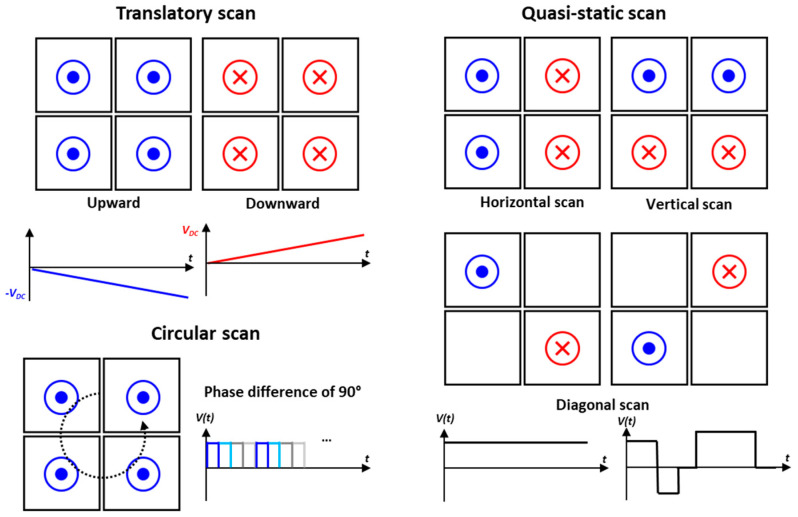
Examples of capable scanning strategies using quasi-static actuation.

Figure 8 shows the quasi-static driving methods to generate various scan patterns. Firstly, there is a bidirectional translatory scan where moving upward and downward can be realized by linearly increasing or decreasing the voltage level applied on the four piezo actuators. The blue and red colored notation mean negative and positive DC voltages, respectively. This is the in-phase movement and there is no phase difference between each piezo actuator. There is also the quasi-static scan, where tilting the mirror plate along horizontal, vertical, and diagonal can be achieved by applying the DC voltage as shown in Figure 8. When using all four actuators, it is possible to reach the maximum scan angle, but it is also feasible to utilize only two of them. By adjusting the magnitude of the applied voltage, it makes it possible to control the tilting angle of the mirror plate and obtain vector graphics by controlling the magnitude and timing of the voltage applied to each plate. For the circular scan enabling the 360° of omnidirectional scanning, it is available by sequentially applying the voltage on each piezo actuator with a phase difference of 90°. For all quasi-static scans, aside from the DC voltage, low frequency in the range of a few to few tens hertz can also be utilized with various waveforms including sinusoidal, sawtooth, and square wave.

### 3.3. Resonant Driving

**Figure 9:**
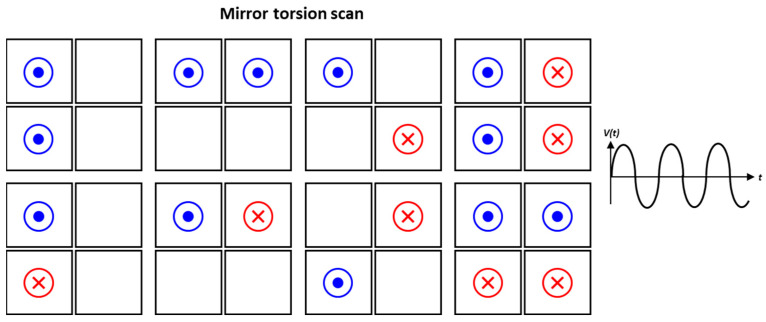
Examples of 1D mirror torsion scanning mode.

Compared to quasi-static driving, the applied voltage signal is a sinusoidal waveform for the resonant driving method. Figure 9 shows the possible combinations of utilizing piezo actuators to obtain the 1D mirror torsion scanning mode. Indicators of the same color represent in-phase actuation, while different colors indicate 180° phase difference of out-of-phase actuation. Likewise, it is possible to use all four piezo actuators for driving, but using two for actuation and the other two as sensors for scan angle detection is also possible. Particularly, when utilizing resonant modes, determining the combination to use is closely related to the mode shape in resonance. In this case, using the two piezo actuators located diagonally (Q1Q3 or Q2Q4 pair) is the effective method among various combinations.

**Figure 10:**
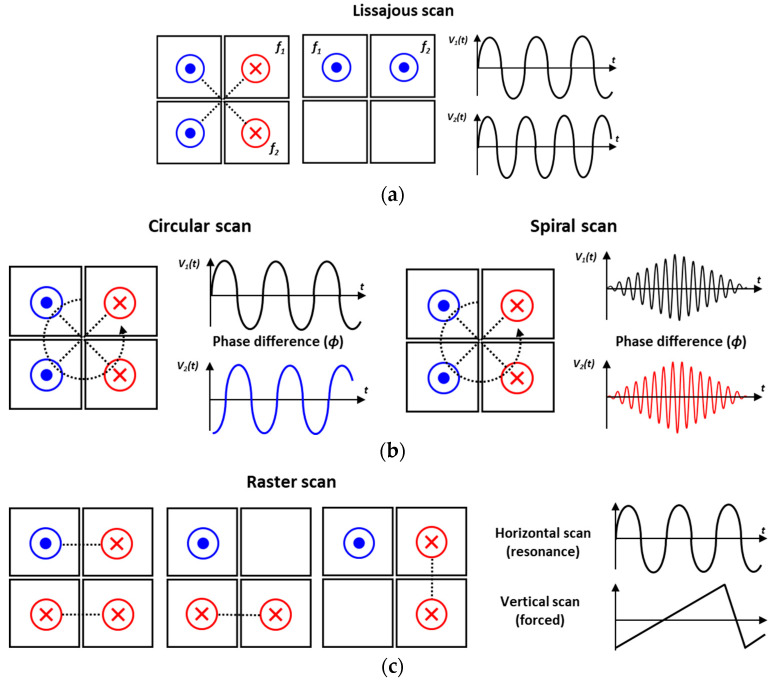
Some 2D scan examples of (**a**) Lissajous, (**b**) circular and spiral, and (**c**) raster scan modes.

Available 2D scan patterns and illustrative driving methods are shown in Figure 10. The Lissajous scan can be acquired by applying the different frequency voltage signals to the Q1Q3 and Q2Q4 pair, respectively (Figure 10a). Alternatively, another approach involves employing a total of two actuators, while utilizing the remaining two as sensors for scan angle detection. In the case of Lissajous scan pattern, the density of the generated scan pattern can be adjusted depending on the applied driving frequencies and phase difference between two driving signals [50,51].

For circular scans, the same actuator combination of Lissajous scan can be utilized but the same driving frequencies have to be applied to the actuators (Figure 10b). In principle, the phase difference between two driving signals is 90° but, due to cross-coupling between the two scanning modes, the actual required phase difference differs in value [47]. A spiral scan pattern is derived from the circular scan (Figure 10b). It is characterized by a gradual increase or decrease of a circular scan pattern and can be achieved by utilizing the amplitude modulated signals with phase difference as a compensation for cross-coupling between adjacent spring structures. A raster scan can be considered as a combination of resonant and quasi-static modes. For resonant mode, it is typically named horizontal scan mode with fast scanning frequency and a sinusoidal signal is utilized. Quasi-static actuation is used for vertical scans (forced actuation) and sawtooth or ramp signals are utilized to return from the ending point of the scan pattern to the starting point [29]. With a combination of four plates and various types of voltage signals, this MEMS mirror device is allowed for implementing a wide range of scan patterns and thus scanning strategies that can be applied to multiple applications.

## 4. Device Characterization and Various Scanning Strategies

### 4.1. Optical Measurement Setup

**Figure 11:**
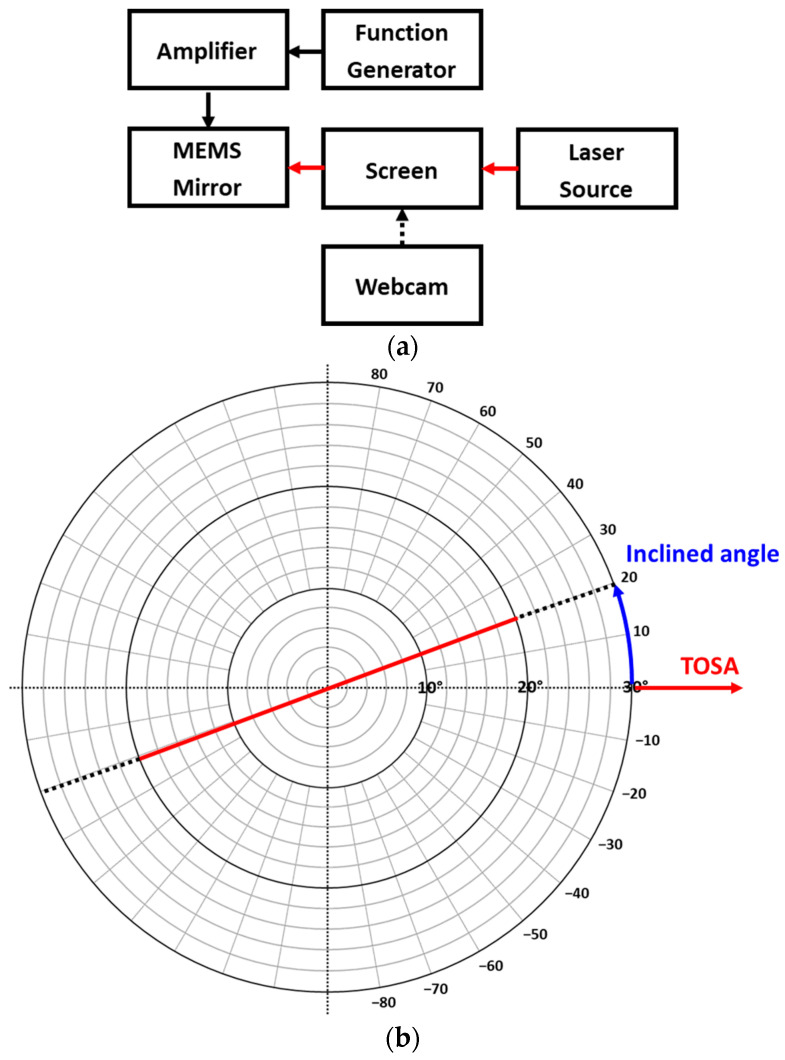
Designed optical measurement setup. (**a**) Block diagram and (**b**) utilized screen displaying predefined TOSA and inclined angle.

The designed AlScN MEMS mirror has been characterized by an optical measurement setup shown in Figure 11. Figure 11a describes the block diagram of characterization setup. The voltage signals including DC, ramp or sawtooth, and sinusoidal waveforms were generated with the function generator (Agilent 33500B, Keysight, Santa Rosa, CA, USA) and applied to the x20 amplifier (High Voltage Amplifier WMA-02, Falco Systems, TH Katwijk aan Zee, The Netherlands) to generate the voltage up to 40 V_pp_. The amplified voltage signals were applied on the piezo actuators and drove the piezoelectric MEMS mirror. A 650 nm laser beam (FLEXPOINT FP-D-650-1D-C-F, LASER COMPONENTS GmbH, Olching, Germany) passed through the screen and was incident on the mirror plate at a 90° angle, and on the opposite side of the screen, a webcam was positioned to monitor the real-time changes in the scan pattern. The distance between the screen and the laser source was 30 cm. The newly designed screen displaying predefined total optical scan angle and inclined angle for the characterization of the MEMS mirror is shown in Figure 11b.

### 4.2. Quasi-Static Characterization

**Figure 12:**
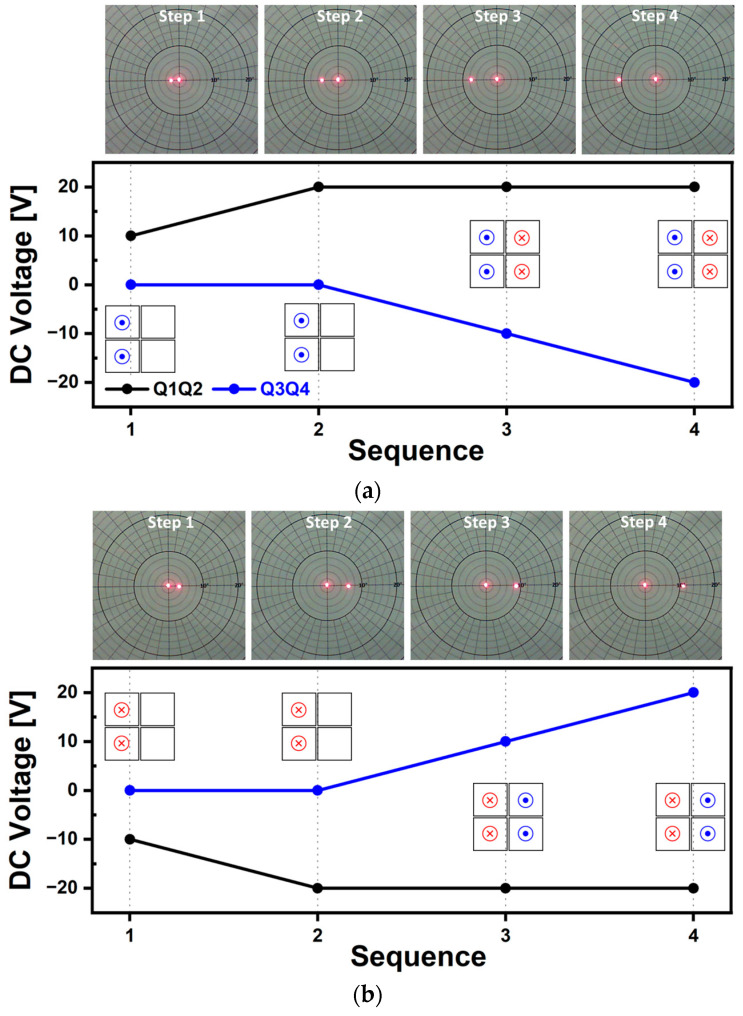
Quasi-static characterization results of horizontal scan capability. Photographs and utilized voltage sequence for mirror tilting in the (**a**) left and (**b**) right directions.

**Figure 13:**
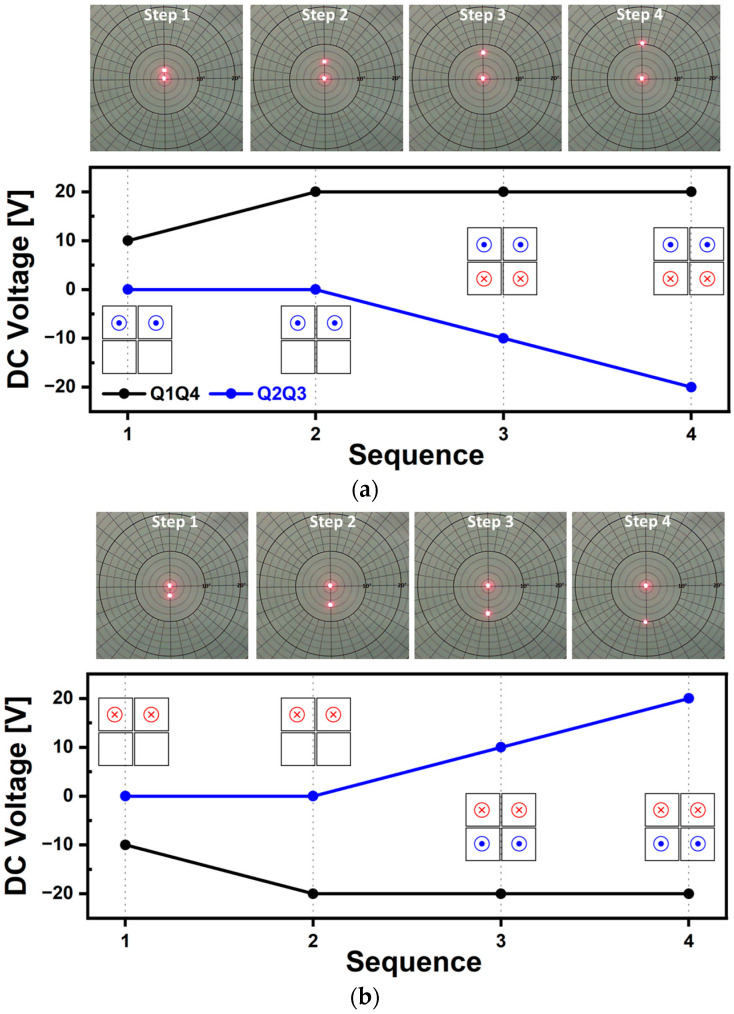
Quasi-static characterization results of vertical scan capability. Photographs and utilized voltage sequence for mirror tilting in the (**a**) upward and (**b**) downward directions.

**Figure 14:**
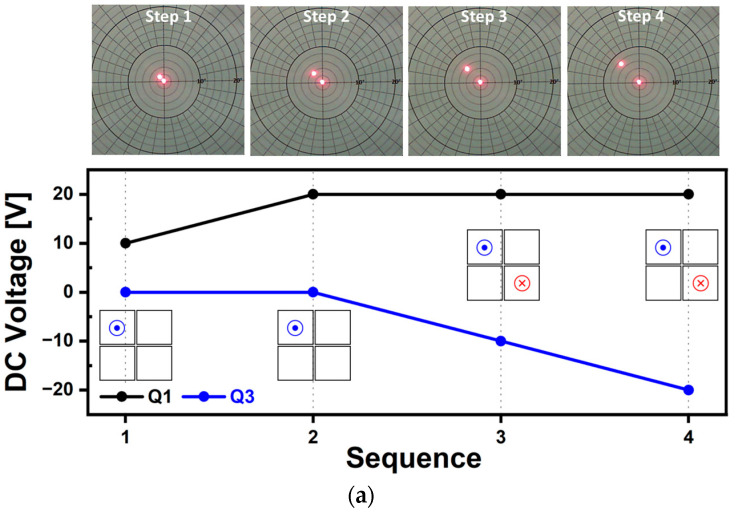

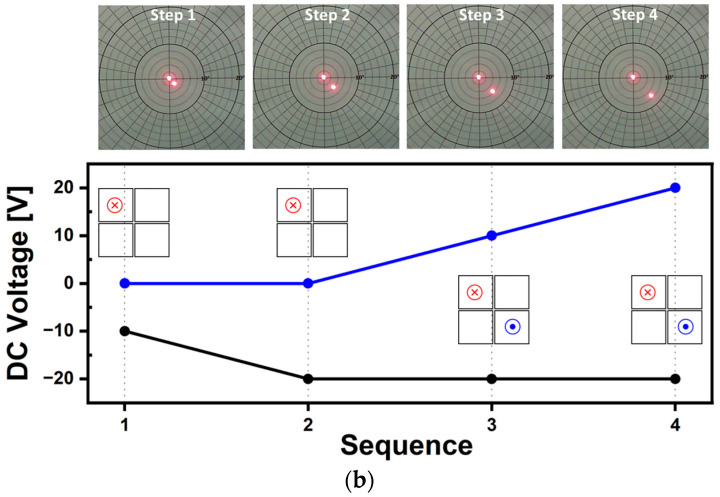
Quasi-static characterization results of diagonal scan capability (1). Photographs and utilized voltage sequence for mirror tilting in the (**a**) upward of +135° and (**b**) downward of −45° directions.

**Figure 15:**
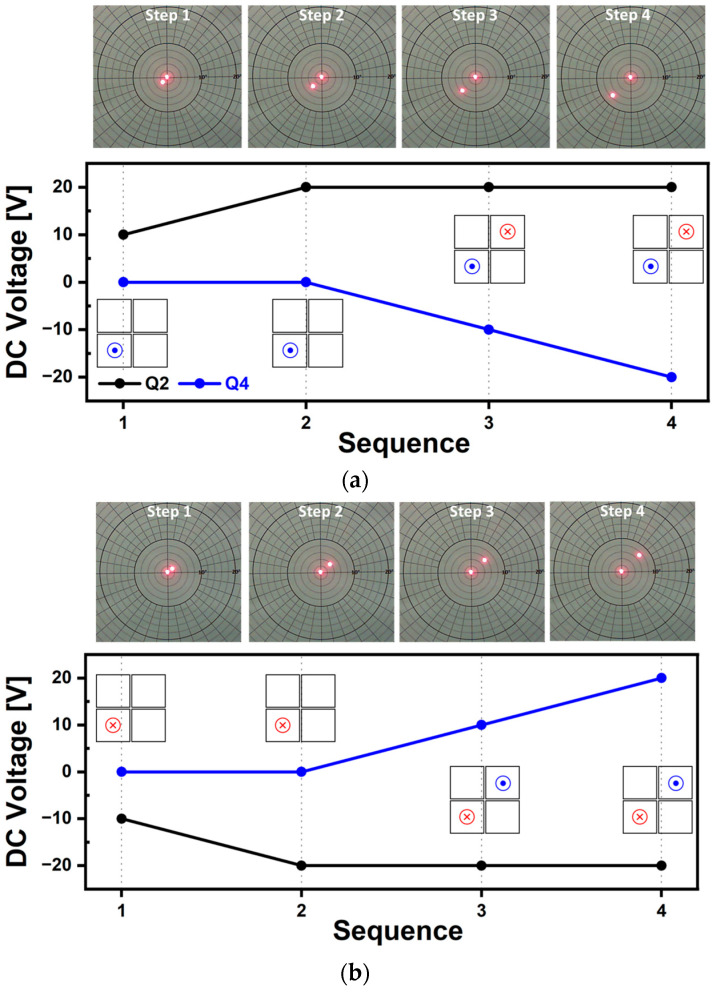
Quasi-static characterization results of diagonal scan capability (2). Photographs and utilized voltage sequence for mirror tilting in the (**a**) downward of −135° and (**b**) downward of +45° directions.

Quasi-static scanning capabilities along various scanning directions of horizontal, vertical, and diagonal axis were tested and the resultant laser beam trajectories are shown in Figure 11, Figure 12, Figure 13 and Figure 14, respectively. The voltage range utilized was from −20 V to 20 V, with DC voltages increased by 10 V by each experimental step. Based on Figure 12a, the testing sequence can be described as follows: In Sequence 1, a positive voltage of 10 V was applied to Q1 and Q2 and as a result, both piezo actuators bent in the downward direction, causing the mirror plate to tilt towards the left. In Sequence 2, an increased voltage of 20 V was applied and the tilting amount of mirror plate was doubled. During Sequence 3, a negative voltage of 10 V with opposite polarity was applied to Q3 and Q4, causing both actuators to bend in the upward direction. By doing so, the degree of mirror tilting can be further increased. The voltage magnitudes and polarities applied to each actuator were changed step by step. As a result, under the voltage range of ±20 V, in Sequence 4, the mirror plate tilted up to a maximum scan angle of 5.2° in the leftward direction. Figure 12b shows the resultant laser beam trajectories by using the voltage with an opposite polarity. Overall, the results shown in Figure 12 show that the tilting level of the designed MEMS mirror can be controlled by utilizing a DC voltage range of ±20 V, within the range of ±5.2°. The results of the vertical scan capability test using the same driving methods are presented in Figure 13. The results from Figure 12 and Figure 13 demonstrated that the utilized piezoelectric material of AlScN can bend bidirectionally based on the polarity of the applied voltage, as confirmed experimentally. Additionally, under the same voltage, similar angles of mirror tilting were achievable in both the horizontal and vertical directions and exhibited a linear trend. Diagonal movement was also tested, and in this case, a combination of Q1Q3 as one pair (Figure 14) and Q2Q4 as another pair (Figure 15) was utilized to achieve the results. The obtained scan angle under ±20 V input signal was ±3.7° along the diagonal scanning direction. Based on the quasi-static characterization results, depending on the combination of actuators used, it was possible to move the laser beam incident on the mirror plate to the desired points on a 2D plane by adjusting the magnitude and polarity of the DC voltage.

### 4.3. One-Dimensional Scanning Characterization

**Figure 16:**
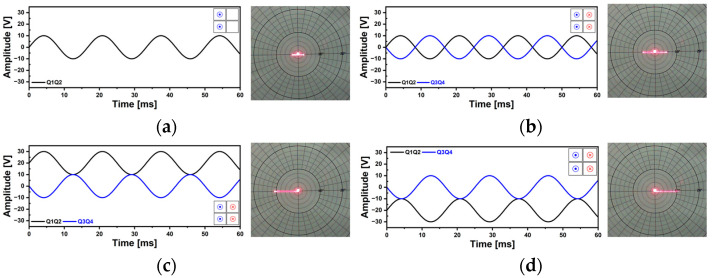
The 60 Hz sinusoidal driving test results. (**a**) Q1Q2 in-phase actuation, (**b**) out-of-phase actuation using Q1Q2 and Q3Q4 combinations, (**c**) with the applied DC offset voltage of 20 V and (**d**) −20 V on Q1Q2 actuators.

One-dimensional line scan characterization can be categorized into non-resonant and resonant operation. Figure 16 shows the 1D line scan trajectories driven at non-resonant frequency of 60 Hz. When a voltage of 20 V_pp_ was applied on the Q1 and Q2 piezo actuator, a TOSA of 3° was obtained as shown in Figure 16a. Figure 16b shows the out-of-phase driving method where the additional driving signal having 180° phase difference was applied on the Q3 and Q4 actuator. This out-of-phase actuation enhanced the tilting amount of mirror plate and the resultant line scan was doubled. Figure 16c,d shows the possibility to change the location of the generated line scan by adjusting the level of offset voltage. When the offset voltages of +20 V and −20 V were additionally applied to the Q1 and Q2 actuators, the generated 1D scanning line was shifted to the left (Figure 16c) and right (Figure 16d) direction. This driving method can be utilized to move the fixed lines with various driving or scanning frequencies to a desired range.

**Figure 17:**
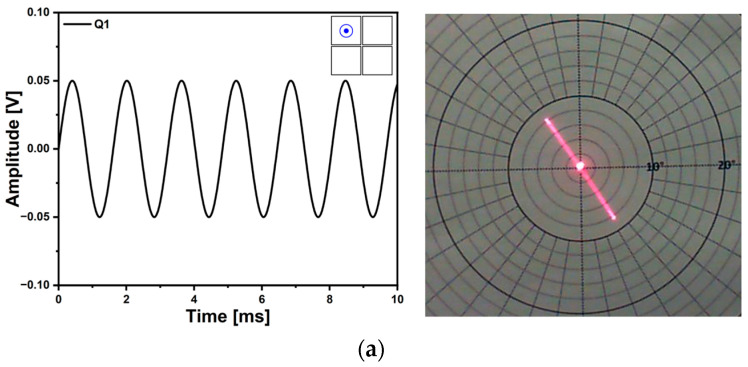

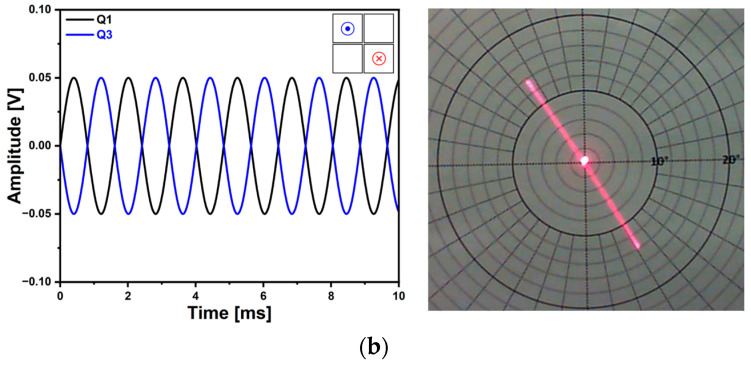
One-dimensional laser beam scan trajectories under resonant operation. (**a**) Single actuation using Q1 and (**b**) out-of-phase actuation using two piezo actuators of Q1 and Q3.

Using resonant operation, a 1D line scan can be achieved under a low level of voltage but unlike the non-resonant driving, the scanning frequency is pre-determined with the designed structure. The resultant 1D line scan trajectories and corresponding applied voltage signals are shown in Figure 17. The applied voltage was 100 mV_pp_ and resonant frequency was 620 Hz. Even though only a single actuator of Q1 was utilized, a TOSA of 8° was obtained with the low level of applied voltage as shown in Figure 17a. The performance of the mirror device could further be enhanced by utilizing the out-of-phase actuation method. The phase difference between Q1 and Q3 was 180° and a TOSA of 14° was obtained under the same level of voltage (Figure 17b).

### 4.4. Two-Dimensional Scanning Characterization

**Figure 18:**
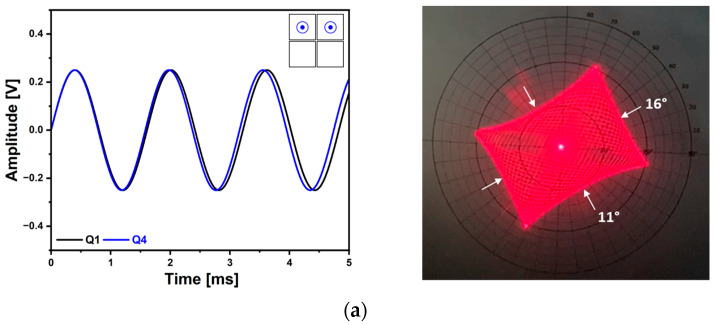

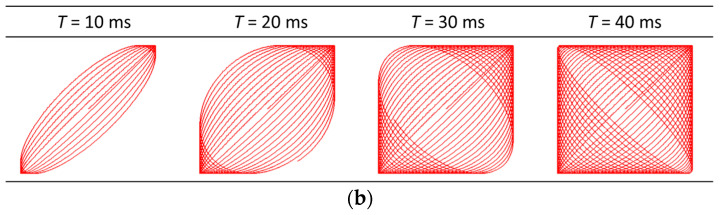
Lissajous scan capability (1). (**a**) Applied voltage signals using Q1 and Q4 piezo actuators and resultant Lissajous scan beam trajectory, (**b**) theoretically plotted graph that illustrates the changes in the scanning pattern over time.

**Figure 19:**
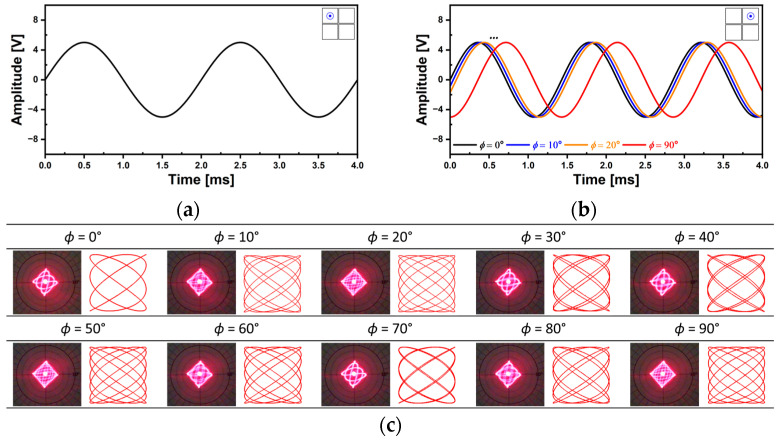
Lissajous scan capability (2). (**a**) Voltage signal applied on Q1 and (**b**) Q2 piezo actuator with phase modulation, (**c**) comparisons between achieved laser beam trajectories and estimated Lissajous scan obtained with the analytic model depending on the change of the phase.

Using the newly developed AlScN MEMS mirror, various 2D scan patterns can be realized. Figure 18 shows the Lissajous scan capability. Two piezo actuators of Q1 and Q4 were utilized and the driving frequencies were 621.3 Hz and 632.5 Hz with the applied voltage of 500 mV_pp_ sinusoidal signals (Figure 18a). The 2D TOSA of 11° × 16° was achieved and the resultant Lissajous scan pattern showed uniform and homogeneous laser beam intensity. Based on the theoretical analysis, the changes of the scan pattern over time were examined (Figure 18b). The scan pattern’s coverage of the region of interest was increased over the time interval of 10 ms and based on theoretical analysis, the resultant scan pattern shown in Figure 18a was obtained at a time of 40 ms elapsed. A Lissajous scan pattern using selected driving frequencies also can be utilized (Figure 19). The utilized driving frequencies were 500 Hz for Q1 and 700 Hz for Q4 piezo actuator and the corresponding voltage signals are shown in Figure 19a and b, respectively. The two driving frequencies were selected because it was possible to realize a stable and stationary Lissajous scan pattern. The applied sinusoidal voltage amplitude was 10 V_pp_. While keeping the driving frequencies and voltage amplitude constant, the changes in Lissajous scan pattern density were observed by incrementally increasing the phase of Q4 by 10° (Figure 19b). The resultant Lissajous scan patterns are shown in Figure 19c and compared with the analytic results. The result implies that the density of Lissajous scan pattern can be controlled by adjusting the phase difference between two driving frequencies while maintaining the 2D field-of-view (FOV) of the corresponding scan pattern.

**Figure 20:**
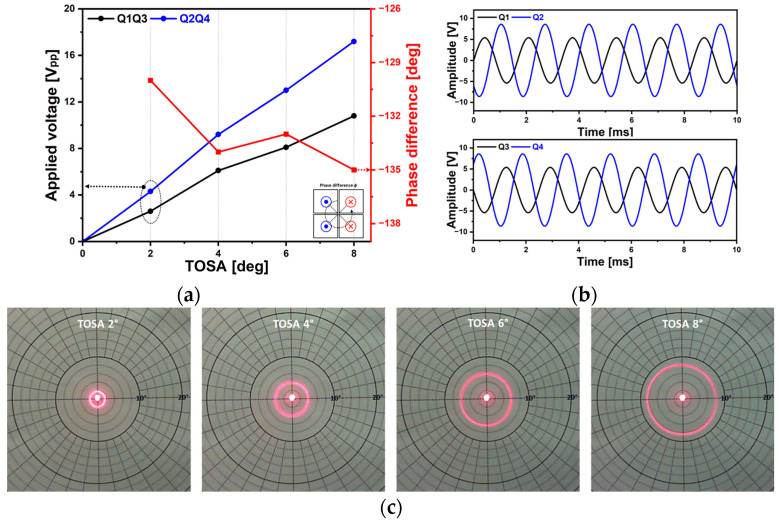
Circular scan capability. (**a**) The applied voltage vs. TOSA with the corresponding phase difference value, (**b**) applied voltage signals to achieve TOSA 8° of circular scan pattern, and (**c**) resultant laser beam trajectories with various size of circular scan pattern.

**Figure 21:**
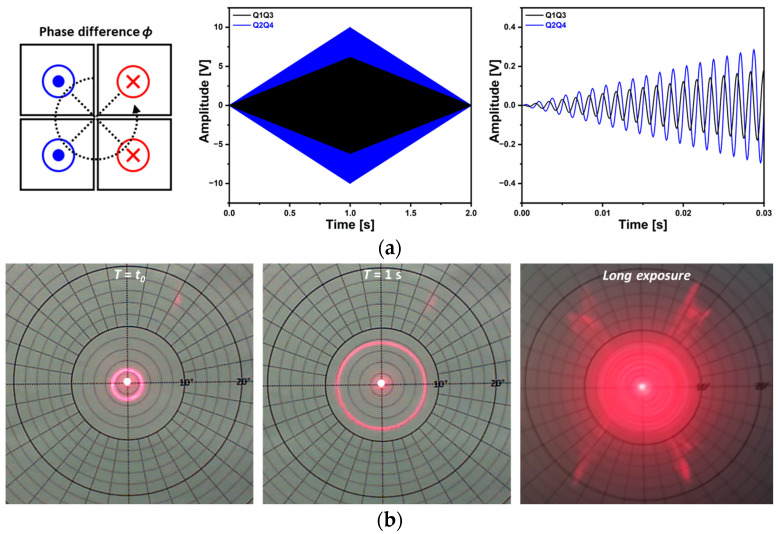
Spiral scan capability. (**a**) The utilized amplitude modulated voltage signal to generate spiral scan, (**b**) resultant circular scan patterns over time and the photograph acquired through long exposure.

Figure 20 shows the circular scan capability of the proposed piezoelectric MEMS mirror. Only two driving signals were utilized to realize the various sizes of circular scan patterns and the frequency was 600 Hz. The required voltages and phase difference between two driving signals for a TOSA of 2° to 8° are shown in Figure 20a. The theoretically required phase difference to generate the wobbling motion of the mirror device is 90° but experimentally measured phase values ranged from −130° to −135° due to the cross-coupling between spring structures. Figure 20b presents the applied voltages to achieve a TOSA of 8° and the resultant circular laser beam trajectories are shown in Figure 20c. Spiral scan capability was also tested and shown in Figure 21. The amplitude modulated driving signals were utilized (Figure 21a). The driving frequencies were 600 Hz and 12.3 V_pp_ and 20 V_pp_ of voltages were applied, respectively. The utilized modulation frequency was 0.5 Hz and the phase difference between the two driving signals was −132°. Figure 21b shows the corresponding circular scan patterns over time and the size of the circular scan pattern was successfully changed with the modulated voltage amplitude signal.

**Figure 22:**
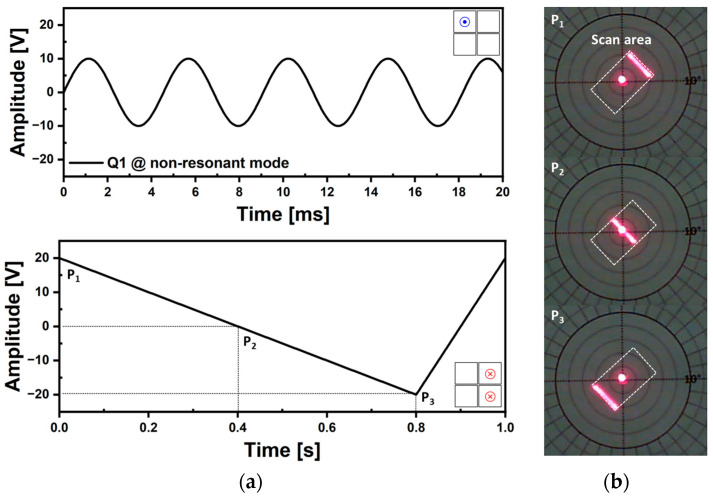
Raster scan capability using non-resonant mode (1). (**a**) The utilized voltage signals of sinusoidal waveform for fast line scanning and ramp waveform (20% symmetry) for forced actuation, (**b**) resultant laser scan trajectories over time.

Raster scanning capabilities have been characterized by two different ways of utilizing non-resonant mode and resonant mode. Figure 22 shows the resultant raster scan pattern using non-resonant mode. The sinusoidal signal of 220 Hz was applied on the Q1 actuator to make a 1D line scan and 1 Hz of ramp signal was utilized for the Q3 and Q4 actuators for forced actuation (Figure 22a). When the voltage amplitude changes from +20 V (point P_1_) to −20 V (point P_3_), the Q3 and Q4 actuators bend upward and the 1D line scan moves downward along the diagonal direction (Figure 22b). This scanning method can be utilized when scanning a region of interest line by line. By adjusting the frequency of the ramp signal, the scanning speed can be controlled, or by modifying the symmetry, it is possible to perform unidirectional line scanning or bidirectional line movement from the first quadrant to the third quadrant at a constant speed (50% symmetry).

**Figure 23:**
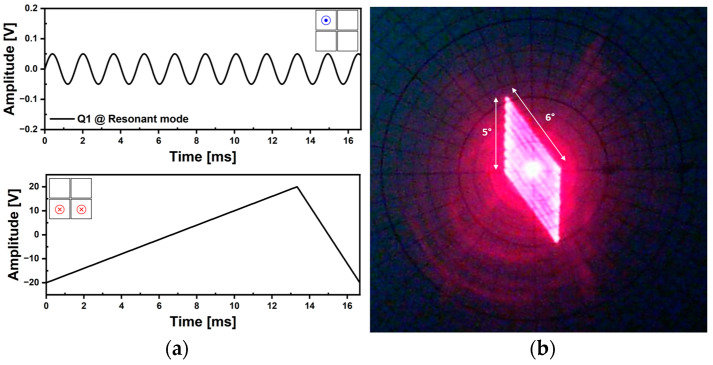
Raster scan capability using resonant mode (2). (**a**) The utilized voltage signals of sinusoidal waveform for fast line scanning and ramp waveform (80% symmetry) for forced actuation, (**b**) resultant laser beam trajectory.

A raster scan realized by using the resonant operation is shown in Figure 23. The Q1 actuator was utilized to drive in resonant mode and the applied voltage amplitude and frequency were 100 mV_pp_ and 619.8 Hz, respectively. Both Q2 and Q3 actuators were utilized for forced actuation and the ramp signal of 60 Hz frequency with 40 V_pp_ voltage amplitude and 80% symmetry was utilized (Figure 23a). The achieved raster scan pattern is shown in Figure 23b and the resultant 2D TOSA is 6° × 5°.

## 5. Discussion and Conclusions

This paper presents the newly developed AlScN piezoelectric MEMS mirror. The proposed 3D constructed MEMS mirror device is compact and can also drive under a relatively low voltage level of ±20 V. Both quasi-static and resonant scan modes were characterized and based on the various piezo actuator combinations, diverse scan patterns including 1D line scan and 2D area scan were realized with the designed MEMS mirror device. Quasi-static actuation along horizontal, vertical, and diagonal directions was characterized and corresponding results show that the tilting angle of the mirror plate can be controlled easily by adjusting the applied DC voltage amplitude. The 1D line scanning results include the non-resonant and resonant driving methods. Non-resonant scanning is advantageous for selecting the desired driving frequency within a few tens of hertz and is also able to shift the location of the generated line scan by adjusting the offset voltage of applied signals. Resonant scanning is a competitive method to reduce the required voltage level and enhance the achievable scan angle. The 2D area scanning methods were also experimentally analyzed. The Lissajous scan pattern was realized by utilizing two actuators and the density of scan pattern can be adjusted by modulating the phase difference between two driving frequencies. The two remaining piezoelectric cantilever structures can also be utilized for scan angle sensors and related study is currently ongoing. The circular and spiral scan patterns were characterized and the results show the capability for realizing omnidirectional scanning with related optics. The line-by-line scanning strategies regarding the raster scan pattern were demonstrated by changing the condition of ramp signals.

In this paper, the presented experimental results were obtained under a relatively low voltage level of ±20 V in order to demonstrate the ability to achieve various scan patterns, without the need for high-voltage circuits. Based on our electrical test results, AlScN can withstand a high voltage of ±120 V, which means that further increases in the TOSA of the mirror device can be expected by increasing the applied voltage level, especially for quasi-static actuation. High-voltage experiments to achieve an enhanced scan angle of more than 20° are ongoing to confirm repeatability. The newly developed AlScN based-piezoelectric MEMS mirrors show multiple scan pattern capabilities and possess the potential to be applied for various scanning strategies and applications.

## Data Availability

The data presented in this study is contained within the article.
